# Hypoglycemic effects of dendrobium officinale leaves

**DOI:** 10.3389/fphar.2023.1163028

**Published:** 2023-06-09

**Authors:** Ming Lv, Qingqing Liang, Xiaofang He, Xiaocui Du, Yuhan Liu, Yan Liu, Chongye Fang

**Affiliations:** ^1^ College of Food Science and Technology, Yunnan Agricultural University, Kunming, China; ^2^ Yunnan Land and Resources Vocational College, Kunming, China; ^3^ Yunnan Vocational College of Mechanical and Electrical Technology, Kunming, China; ^4^ College of Tea Science, Yunnan Agricultural University, Kunming, China; ^5^ Yunnan Research Center for Advanced Tea Processing, Yunnan Agricultural University, Kunming, China; ^6^ International College, Yunnan Agricultural University, Kunming, China

**Keywords:** dendrobium officinale leaves, hypoglycemic, type-2 diabetes (T2D), glucose metabolism, traditional Chinese medicine

## Abstract

**Introduction:** Numerous studies have demonstrated that the stems of *D. officinale* have the effect of lowering blood glucose, but the leaves of *D. officinale* have seldom been investigated. In this study, we mainly studied the hypoglycemic effect and mechanism of *D. officinale* leaves.

**Methods:** Initially *in vivo*, male C57BL/6 mice were administered either standard feed (10 kcal% fat) or high-fat feed (60 kcal% fat) along with either normal drinking water or drinking water containing 5 g/L water extract of *D. officinale* leaves (EDL) for 16 weeks, and changes in body weight, food intake, blood glucose, etc., were monitored weekly. Next *in vitro*, C2C12 myofiber precursor cells which were induced to differentiate into myofibroblasts and cultured with EDL to detect the expression of insulin signaling pathway related proteins. HEPA cells were also cultured with EDL to detect the expression of hepatic gluconeogenesis or hepatic glycogen synthesis related proteins. Eventually after separating the components from EDL by ethanol and 3 kDa ultrafiltration centrifuge tube, we conducted animal experiments using the ethanol-soluble fraction of EDL (ESFE), ethanol-insoluble fraction of EDL (EIFE), ESFE with a molecular weight of >3 kDa (>3 kDa ESFE), and ESFE with a molecular weight of <3 kDa (<3 kDa ESFE) for intensive study.

**Results:** The results *in vivo* revealed that the mice fed the high-fat diet exhibited significantly decreased blood glucose levels and significantly increased glucose tolerance after the EDL treatment, whereas the mice fed the low-fat diet did not. The results *in vitro* showed that EDL activated the expression of protein kinase B (AKT), the phosphorylation of AKT, and the expression of downstream GSK3β in the insulin signaling pathway. EDL treatment of HEPA cells confirmed that EDL did not affect hepatic gluconeogenesis or hepatic glycogen synthesis. In the experiment of studying the composition of EDL, we found that the >3 kDa ESFE displayed the effect of lowering blood glucose. In summary, the effect of EDL in lowering blood glucose may bethanole achieved by activating the insulin signaling pathway to increase insulin sensitivity, and the main functional substance was contained within the >3 kDa ESFE.

**Discussion:** The findings of this study represent a reference point for further exploration of the hypoglycemic effects of *D. officinale* leaves and may assist in both the identification of new molecular mechanisms to improve insulin sensitivity and the isolation of monomeric substances that lower blood glucose. Furthermore, the obtained results may provide a theoretical basis for the development of hypoglycemic drugs with *D. officinale* leaves as the main component.

## 1 Introduction

*Dendrobium officinale* refers to a perennial epiphytic herb belonging to the Dendrobium of Orchidaceae genus that is primarily distributed in the southwestern and southeastern provinces of China. Since ancient times, *D. officinale* has been known as the “gold in medicine”. At least 190 compounds have been isolated from *D. officinale*, including polysaccharides, phenanthrene, dibenzyl group, sugars and glycosides, essential oils, alkaloids, and other species. There are five main classes of chemicals in *D. officinale*: polysaccharides, dibenzyl compounds and phenanthrene compounds, alkaloids, amino acids, and trace elements and flavonoids (Tang et al., 2017; Ren et al., 2020; Yuan et al., 2020; Chen et al., 2021). The main active ingredient in *D. officinale* is polysaccharides. It is mainly isolated from the stems of Dendrobium officinale with a yield rate of over 30% (Shen et al., 2017).

In recent years, numerous scholars have investigated the pharmacological activity of *D. officinale*, *D. officinale* as a medicinal herbal plant has a long history of being used to attenuate the symptoms of diabetes which is also called “Xiaoke” disease in China. The hypoglycemic efficacy of Dendrobium officinale makes it a common ingredient in Xiaoke decoction for type 2 diabetes treatment (Pang et al., 2015). Dendrobium officinale polysaccharides could decrease the levels of fasting blood glucose, insulin, glycated serum protein, and serum lipid profile and alleviate pancreatic injury as well as the dysregulated metabolism of bile acids and amino acids in type 2 diabetic rats (Chen et al., 2019). In addition, it could regulate the hepatic glucose metabolism via the glucagon-mediated signaling pathways as well as the liver-glycogen structure in HFD/STZ-induced diabetic mice (Liu et al., 2020).

Dendrobium officinale has therapeutic potential in cancer prevention and treatment. Its potential mechanism of action is mainly involved in reducing cancer cell growth and proliferation, triggering apoptosis, and increasing autophagy. Dendrobium officinale could also confer protection against liver injuries and improve liver functions against different forms of liver injuries, such as drug-, chemical-, and acute alcohol-induced injuries and nonalcoholic fatty liver diseases (NAFLD) (Xu et al., 2022). Taken together, these studies indicate that *D. officinale* has a variety of effects, such as antioxidation, hepatoprotective, anticancer, hypoglycemic, anti-fatigue, anti-aging, gastroprotective, and immunomodulatory activities (Tang et al., 2017). Because only the stems of these Dendrobium species are permitted for use according to the Chinese Pharmacopoeia, their leaves are largely discarded.

The global diabetes prevalence in 20–79 year olds in 2021 was estimated to be 10.5% (536.6 million people), rising to 12.2% (783.2 million) in 2045 (Sun et al., 2022). At present, the most commonly used hypoglycemic drugs are metformin, pioglitazone, sulfonylureas, and so on, but their use is associated with many restrictions and can also lead to weight gain, resulting in hypoglycemia and other side effects. As such, new hypoglycemic drugs with fewer side effects and use restrictions are urgently needed. Although *D. officinale* has been confirmed to reduce blood glucose levels, previous studies have primarily focused on the plant stems and the effects of *D. officinale* leaves have rarely been reported. However, *D. officinale* leaves possess chemical constituents similar to those found in stems (Huang et al., 2012; Zhou et al., 2014; Zhang et al., 2017). The biomass of *D. officinale* leaves is considerable, and the ratio of the dry weight of *D. officinale* leaves stems to leaves is approximately 1.6:1 (Zhang et al., 2013; Zeng et al., 2018). Therefore, in this work *D. officinale* leaves were used as the main research object to explore their hypoglycemic effects and obtain insights into the underlying mechanism for further research and development. *Dendrobium officinale* leaves provide a certain theoretical reference for the main components of hypoglycemic drugs.

## 2 Materials and methods

### 2.1 Preparation of water extract of *D. officinale* leaves

*Dendrobium officinale* leaves (500 g) were placed in a heating barrel and tap water (20 L) was added. The resulting mixture was boiled for 30 min then filtered through three layers of medical gauze, repeat twice, and the extracted solution was then poured into a heating barrel for heating and concentration to approximately 1 L. The resulting concentrated solution was allowed to cool then poured into a lyophilization dish, frozen at −80 °C overnight, and lyophilized to obtain the EDL.

The obtained lyophilized water extract was dissolved in ultrapure water (1 L) and absolute ethanol (3 L) was added. The resulting mixture was thoroughly stirred then allowed to stand. The supernatant was removed and centrifuged, and the ESFE and ethanol-insoluble fraction of EDL (EIFE) were lyophilized. The lyophilized ESFE was dissolved in ultrapure water and then transferred to a 3 kDa ultrafiltration centrifuge tube. The sample was centrifuged at 4,000 *g* for 30 min, and then a pipette gun was used to blow up the residual liquid in the upper part of the ultrafiltration tube followed by a second cycle of centrifugation at 4,000 *g* for 30 min. This afforded two fractions with >3 kDa ESFE (upper part of the centrifuge tube) and molecular weight of molecular weight of <3 kDa of ESFE (<3 kDa ESFE) (lower part of the centrifuge tube). Both fractions were lyophilized.

### 2.2 Animals and treatment

The animal experiments used healthy male C57BL/6J mice, Kunming mice, and db/db mice provided by Changzhou Cavens Laboratory Animal Co. Ltd. The mice were maintained in a controlled environment (12-h light/12-h dark cycle, 50%–60% humidity, and 24°C ± 1 °C ambient temperature) and administered standard laboratory food and water *ad libitum*.

After an acclimation period of 1 week, 40 C57BL/6J mice were divided into four groups: low-fat diet + water (LFD + H2O), LFD + water extract of *D. officinale* leaves (LFD + EDL), high-fat diet + water (HFD + H2O), and HFD + EDL, where the EDL dose was 5 g/L. The feeding lasted for 16 weeks.

Another 40 C57BL/6J mice were also divided into four groups: HFD + H2O, HFD + EIFE, HFD + ESFE, and HFD + EDL. The feeding lasted for 4 months. The ESFE dose was 1.8 g/L, and the EIFE dose was 3.2 g/L. The feeding lasted for 16 weeks.

After an acclimation period of 1 week, 18 db/db mice were divided into three groups: normal diet + water (ND + H2O), ND + EDL, and ND + ESFE. The feeding lasted for 16 weeks.

Thirty Kunming mice were divided into three groups: ND + H2O, ND+<3 kDa ESFE, and ND+>3 kDa ESFE. After an acclimation period of 1 week, the body weights of the mice were measured after overnight fasting, and glucose (1 g/kg) plus either the <3 kDa ESFE (7.875 mg/kg) or the >3 kDa ESFE (28.125 mg/kg) were administered by gavage, then the blood glucose levels were measured after 30, 60, 90, and 120 min. The area under the curve (AUC) was calculated by the trapezoidal method. After measured blood glucose levels, we continued to feed the mice with the two components dissolved in water and normal diet for 2 weeks and measure fasting blood glucose every week.

### 2.3 Glucose tolerance test

Glucose tolerance tests play an important role in evaluating insulin resistance. In this work, glucose homeostasis was measured by performing intraperitoneal glucose tolerance tests using the C57BL/6J mice after treatment for 15 weeks. The body weights of the mice were measured after overnight fasting, and a drop of blood was collected from the tail vein to measure the basic blood glucose level (t = 0) using a glucose meter (Roche, Accu-Chek Aviva). Next, the mice were administered 1 g/kg d-glucose (2 mg/g) by intraperitoneal injection and the blood glucose levels were measured at 30, 60, 90, and 120 min. The AUC was calculated by the trapezoidal method.

### 2.4 Cell culture

HEPA and C2C12 cells were used in this study. These cells were cultured in Dulbecco’s modified Eagle’s medium (DMEM/high glucose, Thermo Fisher Scientific) supplemented with 4 mmol/L L-glutamine (supplier) and 10% fetal bovine serum (Biological Industries Israel Beit Haemek Ltd.). DMEM high-glucose medium containing 2% horse serum was used to differentiate muscle fiber cells for 5–7 days. The cells were maintained at 37 °C in a humidified incubator with 5% CO2.

### 2.5 Cell treatment

The water extract of *D. officinale* leaves (10 mg/mL) was added to the differentiated muscle fiber cells and HEPA cells after changing the solution to obtain a final EDL concentration in the culture medium of 0, 50, 100, or 200 μg/mL. The cells were then grown in the incubator for 24 h prior to protein extraction.

### 2.6 Western blotting

Cells or tissue samples were suspended in RIPA buffer (Solarbio, Beijing, China) according to the manufacturer’s protocol. Proteins were separated by 10% SDS-PAGE and transferred onto PVDF membranes (EMD Millipore Corporation, Merck Life Sciences KGaA, Darmstadt, Germany). The membranes were then incubated at 4 °C overnight with various primary antibodies (anti-GSK3β, anti-AKT, anti-P-AKT, anti-P-IR (1,150), anti-GAPDH, anti-PEPCK, anti-G6P, and anti-β-tubulin), followed by the appropriate combination of secondary antibodies according to the manufacturer’s protocols. Images were obtained using a FluorChem E system (ProteinSimple, Santa Clara, CA, United States).

### 2.7 Statistical analysis

All data were analyzed using SPSS 17.0 (IBM Corp., Chicago, IL, United States) and GraphPad Prism 5 (GraphPad Software Inc., La Jolla, CA, United States) for Windows. All values in the text are expressed as the mean ± standard error of the mean. Linear mixed models analysis was used to analyze the body weight data. For other data related to bone metabolism, independent-sample t tests and one-way ANOVA were performed to compare the sham versus model groups and model versus treatment groups using pooled variance, respectively. A probability of *p* < 0.05 was considered significant.

## 3 Results

### 3.1 EDL reduces blood glucose and improves insulin sensitivity in mice fed a high-fat diet

**FIGURE 1:**
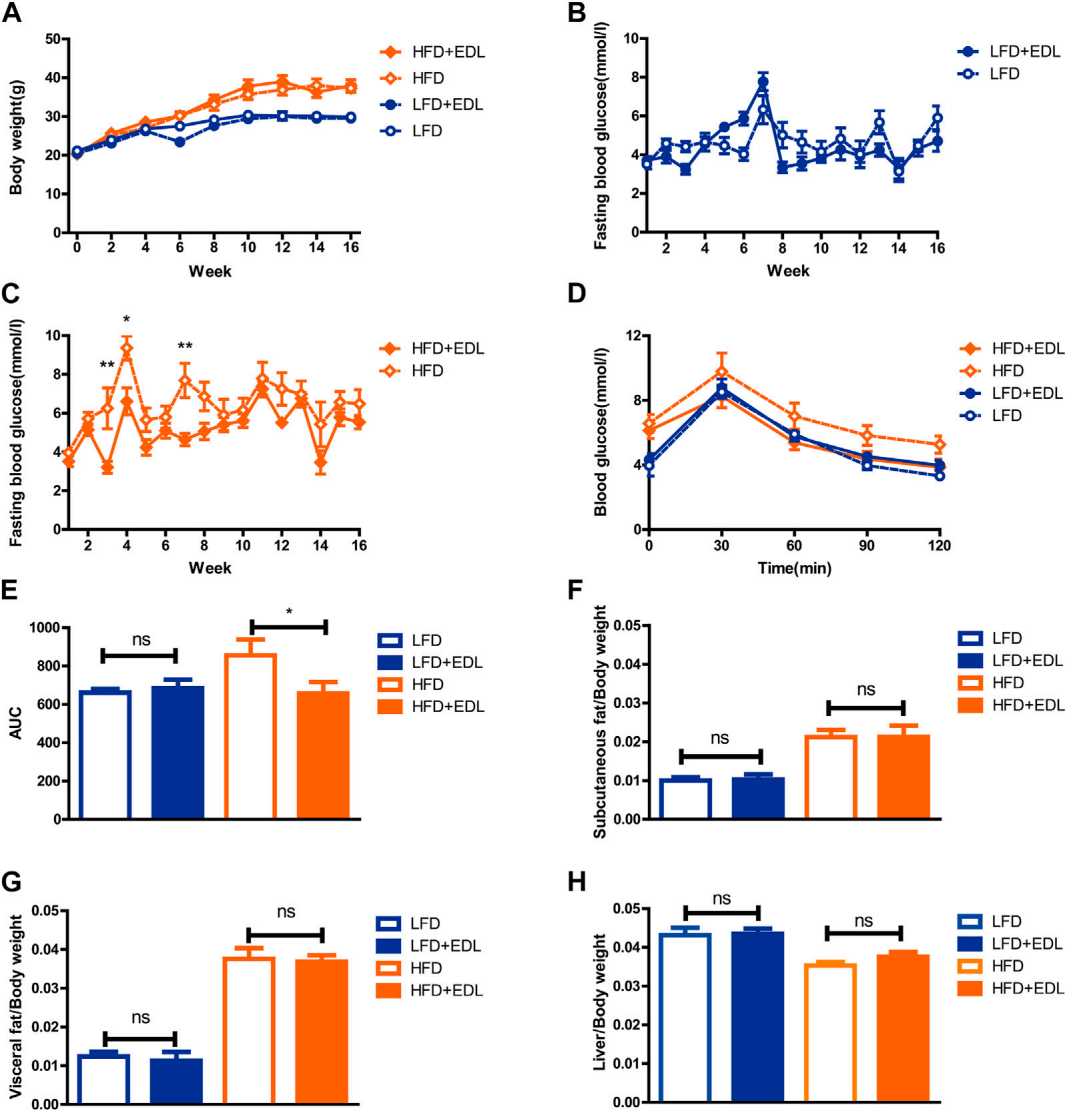
Statistical analysis of body weight, fasting blood glucose, subcutaneous fat, visceral fat, and liver tissue weights, and glucose tolerance in male C57BL/6J mice treated with water extract of *D. officinale* leaves. **(A)** Body weight. **(B)** Fasting blood glucose for the low-fat diet groups. **(C)** Fasting blood glucose for the high-fat diet groups. **(D)** Glucose tolerance. **(E)** Area under the glucose tolerance curve (AUC). **(F)** Subcutaneous fat with respect to body weight. **(G)** Visceral fat with respect to body weight. **(H)** Liver tissue with respect to body weight. Ns, not significant; *p < 0.05; **p < 0.01; ***p < 0.001. LFD:low-fat diet; HFD:high-fat diet; EDL:water extract of **(D)** officinale leaves.

To explore the hypoglycemic effect of EDL, 6–7-week-old male C57BL/6J mice were divided into four groups after acclimation for 1 week. The mice were fed either low-fat or high-fat diets with either sterilized drinking water or drinking water containing 5 g/L EDL (*ad libitum*) for 16 weeks (Fang et al., 2015; Cai et al., 2016). The body weight and blood glucose levels were measured throughout the course of the experiment and the data were collated. The results showed that EDL had no significant effect on the body weights of the mice fed either high-fat or low-fat diets (Figure 1A). In addition, EDL had no significant effect on the fasting blood glucose for the low-fat diet groups (Figure 1B). By contrast, for the high-fat diet groups, the fasting blood glucose was significantly lower for the mice administered EDL-containing drinking water (Figure 1C). At the 15th week, we performed glucose tolerance tests. The results showed that EDL administration had little effect on the glucose tolerance for the low-fat diet groups, whereas it significantly reduced the blood glucose 30 min after injection for the high-fat diet groups (Figure 1D). The area under the glucose tolerance curve was also measured, which revealed no significant difference upon EDL administration for the low-fat diet groups but a significant decrease upon EDL administration for the high-fat diet groups (Figure 1E). These results were consistent with those shown in Figure 1D, indicating that EDL increased the glucose tolerance and improved the intrinsic insulin sensitivity in mice fed a high-fat diet.

After 16 weeks, the mice were euthanized and dissected. The subcutaneous fat, visceral fat, and livers of the mice were removed and weighed. The results revealed that EDL administration did not significantly affect the relative weights of subcutaneous fat, visceral fat, and liver tissue for either the low-fat or high-fat diet groups (Figures 1F–H), in accordance with the total body weight results shown in Figure 1A. It was concluded that the addition of EDL had no significant effect on either the body weight or the relative subcutaneous fat, visceral fat, and liver tissue weights of male C57BL/6J mice for both the low-fat and high-fat diet groups, although it significantly reduced the fasting blood glucose and improved the glucose tolerance and endogenous insulin sensitivity in the mice fed a high-fat diet.

### 3.2 EDL regulates the expression of proteins related to glucose metabolism in the muscles of mice fed a high-fat diet

**FIGURE 2:**
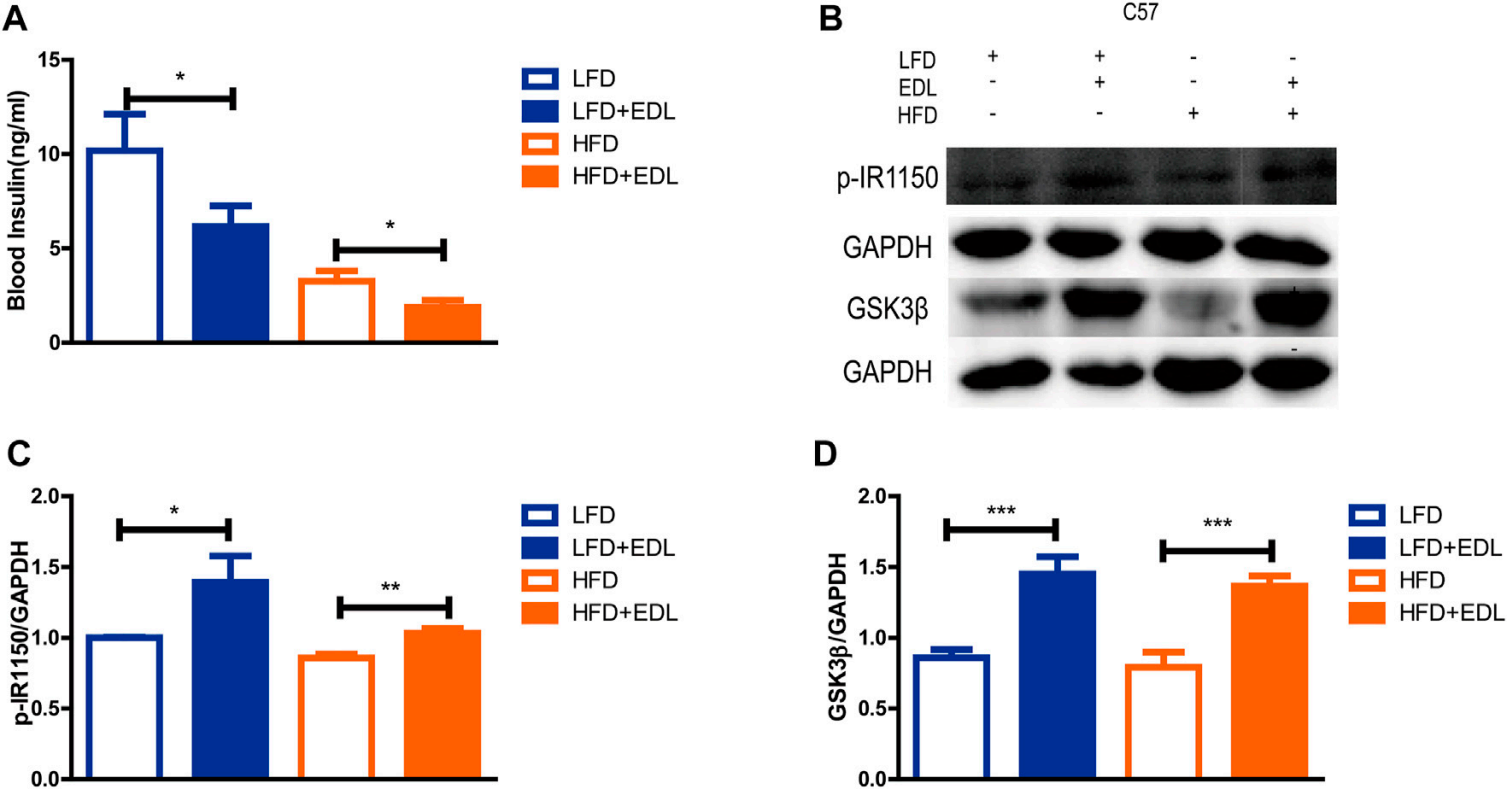
Statistical analysis of blood insulin concentration and muscle p-IR1150 and GSK3β protein contents in male C57BL/6J mice treated with water extract of *D. officinale* leaves. **(A)** Blood insulin concentration. **(B)** Western blots showing the expression of p-IR1150 and GSK3β proteins in muscle tissue. **(C)** Expression of p-IR1150 in muscle tissue. **(D)** Expression of GSK3β in muscle tissue. *p < 0.05, **p < 0.01, ***p < 0.001.

Muscle tissue is known to be an important target organ of insulin, and GSK3β is a key downstream protein of the insulin signaling pathway that plays a role in muscle glycogen synthesis (Cross et al., 1995; Welsh et al., 1996; Srivastava and Pandey, 1998). p-IR1150 is the key upstream node of the insulin signaling pathway (White et al., 1985; White et al., 1988). To examine the specific mechanism of action of EDL, the mice fed the various diets for 16 weeks were euthanized, their thigh muscles were dissected, and the muscle proteins were extracted for Western blotting. The results revealed significantly increased expression of the GSK3β and p-IR1150 proteins in the muscles of the mice treated with EDL for the both the low-fat and high-fat diet groups (Figures 2B–D), indicating activation of the insulin signaling pathway. It is worthwhile to note that mice treated with EDL exhibited significantly lower blood insulin levels (Figure 2A). These results suggest that lower insulin levels were able to mediate greater effects.

### 3.3 EDL regulates the expression of proteins related to glucose metabolism in C2C12 cells

**FIGURE 3:**
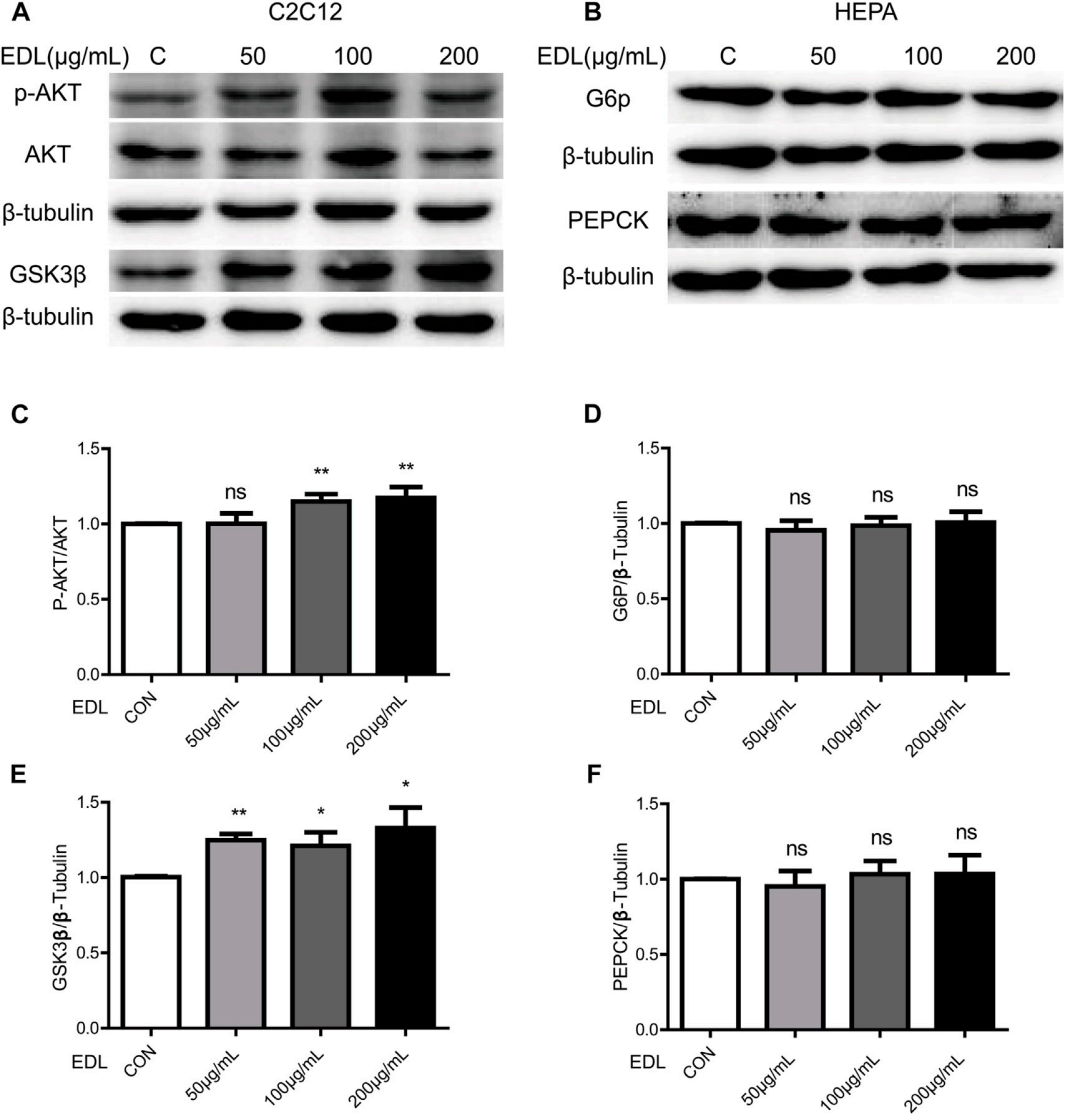
Statistical analysis of the expression of proteins related to the insulin signaling pathway and glucose metabolism in wild-type C2C12 cells and HEPA cells treated with water extract of *D. officinale* leaves. **(A)** Expression of AKT and GSK3β proteins in wild-type C2C12 cells after treatment with water extract of *D. officinale* leaves. **(B)** Expression of G6P and PEPCK proteins in wild-type HEPA cells treated with water extract of *D. officinale* leaves. **(C)** Expression of p-AKT protein in wild-type C2C12 cells after treatment with water extract of *D. officinale* leaves. **(D)** Expression of G6P protein in wild-type HEPA cells treated with water extract of *D. officinale* leaves. **(E)** Expression of GSK3β protein in wild-type C2C12 cells after treatment with water extract of *D. officinale* leaves. **(F)** Expression of PEPCK protein in wild-type HEPA cells treated with water extract of *D. officinale* leaves. Ns, not significant; *p < 0.05; **p < 0.01; ***p < 0.001.

The results described in the previous subsection suggested that EDL reduces fasting blood glucose levels and improves glucose tolerance in mice fed a high-fat diet by activating the insulin signaling pathway. The next step was to conduct *in vitro* experiments with myofibroblasts differentiated from C2C12 cells. We thus measured the expression and phosphorylation of AKT in myofibroblast cells after treatment with various concentrations of EDL (Cross et al., 1995; Hajduch et al., 2001). The activation level of akt is determined by the ratio of p-AKT to AKT expression. The results revealed that the activation level increased with increasing EDL concentration in a concentration-dependent manner (Figure 3C). The similar increase in GSK3β expression suggests that the insulin signaling pathway may be activated in myofibroblasts (Figures 3A,E). In addition, HEPA cells were treated with EDL. The results showed no significant changes in G6P (Au et al., 2000) or PEPCK (Rodgers et al., 2005) expression with increasing EDL concentration (Figures 3B,D,F). These results suggest that the decrease in fasting blood glucose and improved glucose tolerance in mice administered EDL were not attributable to hepatic glycogen synthesis.

### 3.4 ESFE may be the main contributor to reducing blood glucose

**FIGURE 4:**
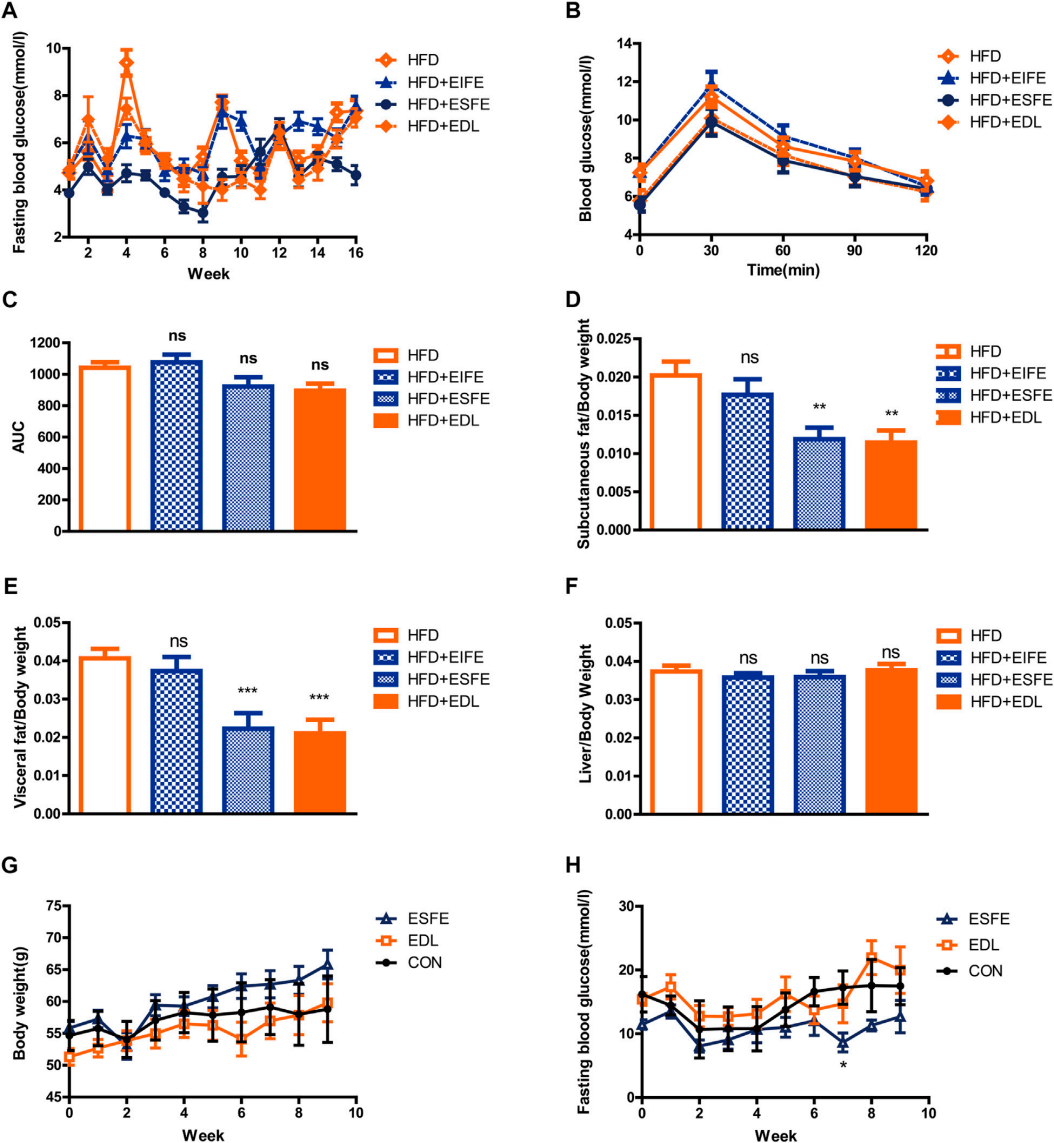
Statistical analysis of fasting blood glucose, subcutaneous fat, visceral fat, and liver tissue contents, and glucose tolerance in male C57BL/6J and db/db mice treated with EDL and its various fractions. **(A)** Fasting blood glucose in C57BL/6J mice. **(B)** Glucose tolerance in C57BL/6J mice. **(C)** Area under the glucose tolerance curve (AUC). **(D)** Subcutaneous fat with respect to body weight in C57BL/6J mice. **(E)** Visceral fat with respect to body weight in C57BL/6J mice. **(F)** Liver tissue with respect to body weight in C57BL/6J mice. **(G)** Body weight for db/db mice. **(H)** Fasting blood glucose in db/db mice. Ns, not significant; *p < 0.05; **p < 0.01; ***p < 0.001. ESFE: ethanol-soluble fraction of EDL; EIFE: ethanol-insoluble fraction of EDL.

Earlier in this section, we demonstrated that EDL reduced the fasting blood glucose and improved glucose tolerance in mice fed a high-fat diet. In an effort to determine the main components of EDL responsible for these effects, we used ethanol to separate the isolated EDL into an ESFE and an EIFE. Male C57BL/6J mice (n = 40) were then divided into four groups and treated with water, EIFE, ESFE, or EDL alongside a high-fat diet while monitoring their fasting blood glucose and glucose tolerance. The results revealed that treatment with ESFE or EDL led to a reduction in the fasting blood glucose compared with the normal drinking water treatment group, whereas treatment with EIFE did not (Figure 4A). Similarly, the glucose tolerance experiments demonstrated that the blood glucose concentration decreased 30 min after injection for the mice in the EDL and ESFE groups compared with those in the control group, indicating that EDL and ESFE improved the glucose tolerance of mice fed a high-fat diet (Figure 4B). Figure 4C shows the areas under the glucose tolerance curves, which were lower for the mice in the EDL and ESFE groups, further indicating that EDL and ESFE promoted improved glucose tolerance in mice fed a high-fat diet. After 16 weeks, the subcutaneous fat, visceral fat, and liver tissue were weighed. The results showed no significant changes in the relative weight of liver tissue for the EDL and ESFE groups compared with the control (Figure 4F), whereas the relative weights of subcutaneous fat and visceral fat were significantly reduced (Figures 4D,E). In order to study whether ESFE still works under hyperglycemia, we examined the effect of ESFE on db/db mice, and the results revealed that ESFE inhibited the blood glucose increase with increasing body weight (Figures 4G,H). These results further confirmed that ESFE reduced blood glucose.

### 3.5 The main component responsible for reducing blood glucose is > 3 kDa ESFE

**FIGURE 5:**
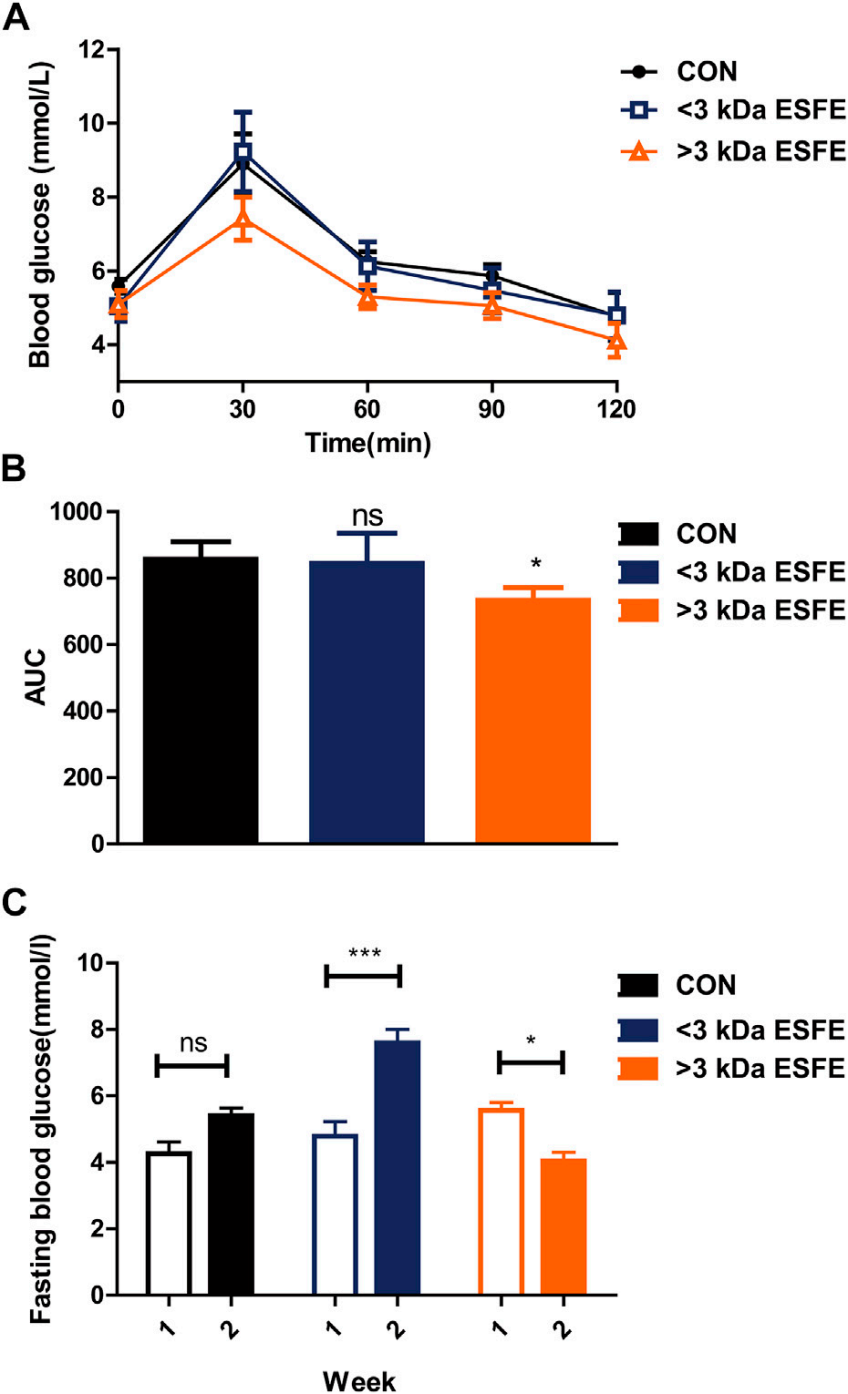
Statistical analysis of glucose tolerance and fasting blood glucose for male Kunming mice treated with the <3 kDa and >3 kDa ethanol-soluble fractions of EDL. **(A)** Oral glucose tolerance. **(B)** Area under the glucose tolerance curve (AUC). **(C)** Fasting blood glucose over 1–3 weeks ns, not significant; *p < 0.05; **p < 0.01; ***p < 0.001. >3 kDa ESFE: EFSE with a molecular weight of >3 kDa; <3 kDa ESFE: ESFE with a molecular weight of <3 kDa.

The results described in the previous subsection indicated that the ESFE fraction of EDL contained the main component responsible for reducing blood glucose. We considered the possibility of isolating the active species on the basis of molecular weight and thus passed the ESFE fraction through a 3 kDa ultrafiltration tube to separate the constituents into a <3 kDa ESFE and a >3 kDa ESFE. These two fractions were separately administered to Kunming mice along with glucose by gavage. The results revealed that the >3 kDa ESFE significantly decreased the blood glucose measured 30 min after administration, which was confirmed by a significant decrease in the area under the blood glucose curve (Figures 5A,B). Next, we continued to feed the mice with the two components dissolved in water and normal diet for 2 weeks. The results confirmed that the >3 kDa ESFE significantly inhibited the increase in blood glucose in Kunming mice (Figure 5C). On the basis of these results, we conclude that the active component responsible for the hypoglycemic effect of the water extract of *D. officinale* leaves was contained mainly in the >3 kDa ESFE.

## 4 Discussion

At present, common hypoglycemic drugs include metformin, pioglitazone, sulfonylureas, and so on. Metformin is the primary drug recommended for the treatment of type 2 diabetes. It decreases insulin resistance and hepatic gluconeogenesis, reducing the glucose concentration without increasing the risk of hypoglycemia. Because metformin is excreted via the urine, a good glomerular filtration rate is needed (Inzucchi et al., 2014). Moreover, metformin should be used cautiously in patients with congestive heart failure or liver dysfunction owing to the risk of lactic acidosis (American Diabetes, 2019). Pioglitazone is an insulin sensitizer that acts at the transcription level and is characterized by good results, low cost, and no risk of hypoglycemia when used as a monotherapy. It can even be used with poor glomerular filtration rates and is safe for patients with cardiovascular disease (Schneider et al., 2008). However, pioglitazone is associated with weight gain and fluid retention and is therefore contraindicated in the case of congestive heart failure. In addition, the use of this drug in older people at risk of falling is not recommended because it has been shown to increase the risk of non-osteoporosis fractures (Viscoli et al., 2017), and it should also be avoided in patients with or at a high risk of bladder cancer (Lewis et al., 2015). Sulfonylureas stimulate insulin secretion by promoting the depolarization of *β*-cell membranes. They are highly effective and economical but should be used with extreme caution because of the high risk of hypoglycemia and weight gain. Although these compounds are commonly applied as hypoglycemic drugs, their use has numerous limitations and side effects, such that it is crucial to develop new hypoglycemic drugs with fewer side effects and use restrictions.

In this regard, *D. officinale* has been demonstrated to exhibit promising anti-hyperglycemia activity, such as not significantly reducing blood glucose and insulin levels in normal mice while increasing serum insulin levels and reducing serum glucagon levels in streptozotocin-induced diabetic rats. Furthermore, it has been reported to reduce serum glucose levels and increase hepatic glycogen levels in epinephrine-induced hyperglycemic mice (Wu et al., 2004). Among the varieties of *D. officinale*, *D. officinale* has the best hypoglycemic effect (Pan et al., 2014).

In traditional Chinese medicine, *D. officinale* preparations are primarily prepared using the stems of the plant, and most previous research into treating hypoglycemia with *D. officinale* has also focused on the stems. By contrast, the hypoglycemic effects of *D. officinale* leaves have received comparatively little attention. In this study, we thus selected *D. officinale* leaves as the research object to determine whether they also decrease blood glucose levels and explore the underlying mechanism. We first conducted *in vivo* experiments in a mouse model to study the potential hypoglycemic effects of EDL treatment. During 16 weeks of feeding, we observed no significant hypoglycemic effect in mice fed a low-fat diet, whereas in mice fed a high-fat diet there was a decrease in blood glucose. This is similar to the results reported in previous studies for the hypoglycemic effects of *D. officinale* stems. Glucose tolerance tests of mice fed a high-fat diet revealed that EDL significantly improved the glucose tolerance and enhanced endogenous insulin sensitivity. Analysis of the protein expression levels in muscle tissue suggested that EDL mediates this effect by activating the insulin signaling pathway, although EDL also appeared to reduce the plasma insulin levels. These findings indicate that EDL improves insulin sensitivity while reducing the plasma insulin concentration, thus making plasma insulin more effective at lower levels. *In vitro* analysis of protein expression in myofibroblasts also confirmed that EDL activated the insulin signaling pathway. Meanwhile, the results of treating HEPA cells with various concentrations of EDL indicated no effect on hepatic gluconeogenesis and hepatic glycogen synthesis. Next, we separated the components of EDL into two fractions based on their ethanol solubility, namely, the ESFE and the EIFE. Because we had confirmed that EDL displayed no significant hypoglycemic effect in normal mice, we treated mice fed a high-fat diet with EDL, ESFE, or EIFE with water as the control group. After 16 weeks of feeding, we found that treatment with ESFE or EDL reduced blood glucose levels, increased glucose tolerance, and improved insulin sensitivity compared with the control group, whereas treatment with ESFE did not. These findings indicate that the ESFE fraction contained the main active component(s) responsible for the observed effects of EDL.

By confirming ESFE fraction of EDL contained the main component responsible for reducing blood glucose, we separated ESFE fraction with the molecular weight into a <3 kDa ESFE and a >3 kDa ESFE through a 3 k Da ultrafiltration tube. Based on the two very few fractions, we chose Kunming mice for short-term experiment to verify which fraction can decrease blood glucose. After Kunming mice for a week’s rest at the laboratory, we first performed an oral glucose tolerance test to Kunming mice through mixing glucose with a <3 kDa ESFE and a >3 kDa ESFE by Intragastric administration. The results revealed that the >3 kDa ESFE significantly decreased the blood glucose measured 30 min after administration, which was confirmed the >3 kDa ESFE could improve Glucose tolerance. Next, we continued to feed the two components to the mice for 2 weeks. The results confirmed that the >3 kDa ESFE significantly inhibited the increase in blood glucose in Kunming mice. On the basis of the two experiment results, we conclude that the active component responsible for the hypoglycemic effect of EDL was contained mainly in the >3 kDa ESFE.

## Data Availability

The original contributions presented in the study are included in the article/Supplementary Material, further inquiries can be directed to the corresponding author.
