# Supplementary figures and images for: Hypoglycemic effects of dendrobium officinale leaves
Source: Front Pharmacol. 2023 Jun 9;14:1163028. doi: 10.3389/fphar.2023.1163028 (PMC10288155; doi:10.3389/fphar.2023.1163028)

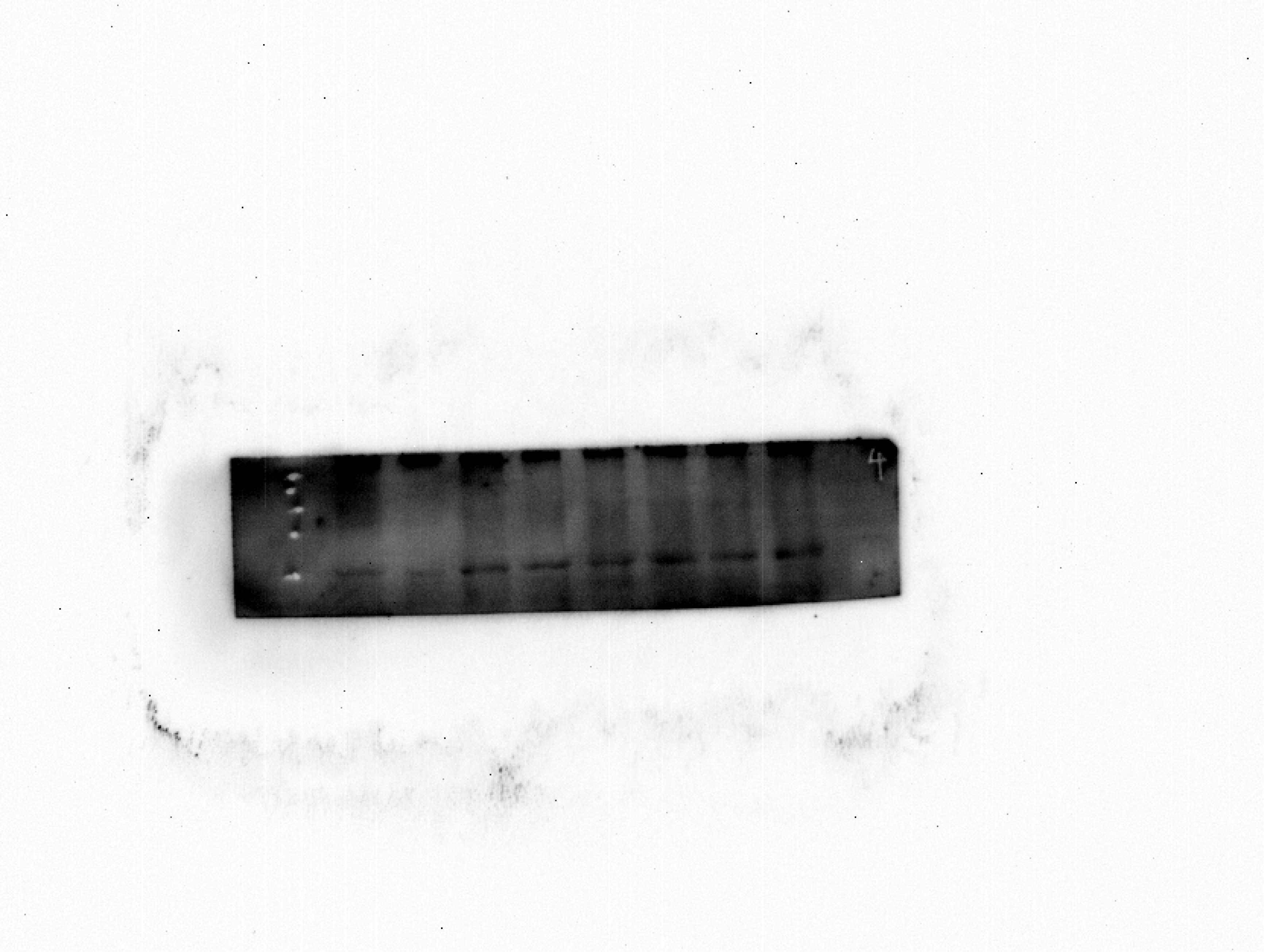

Supplement: Supplementary file 2 [file DataSheet1.ZIP › Westen blot/figure 2/1、p-IR150.tif]

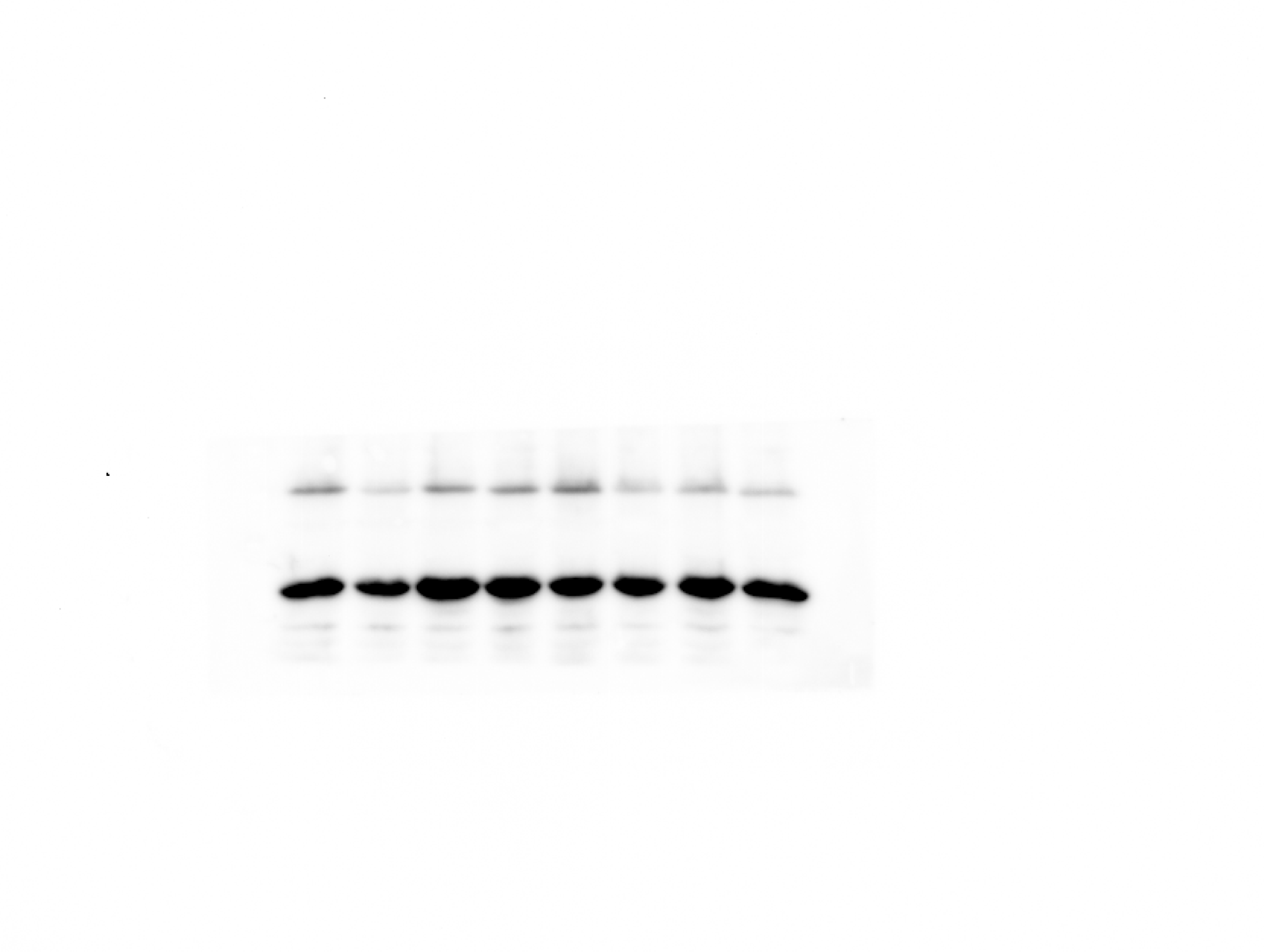

Supplement: Supplementary file 2 [file DataSheet1.ZIP › Westen blot/figure 2/2、GAPDH.tif]

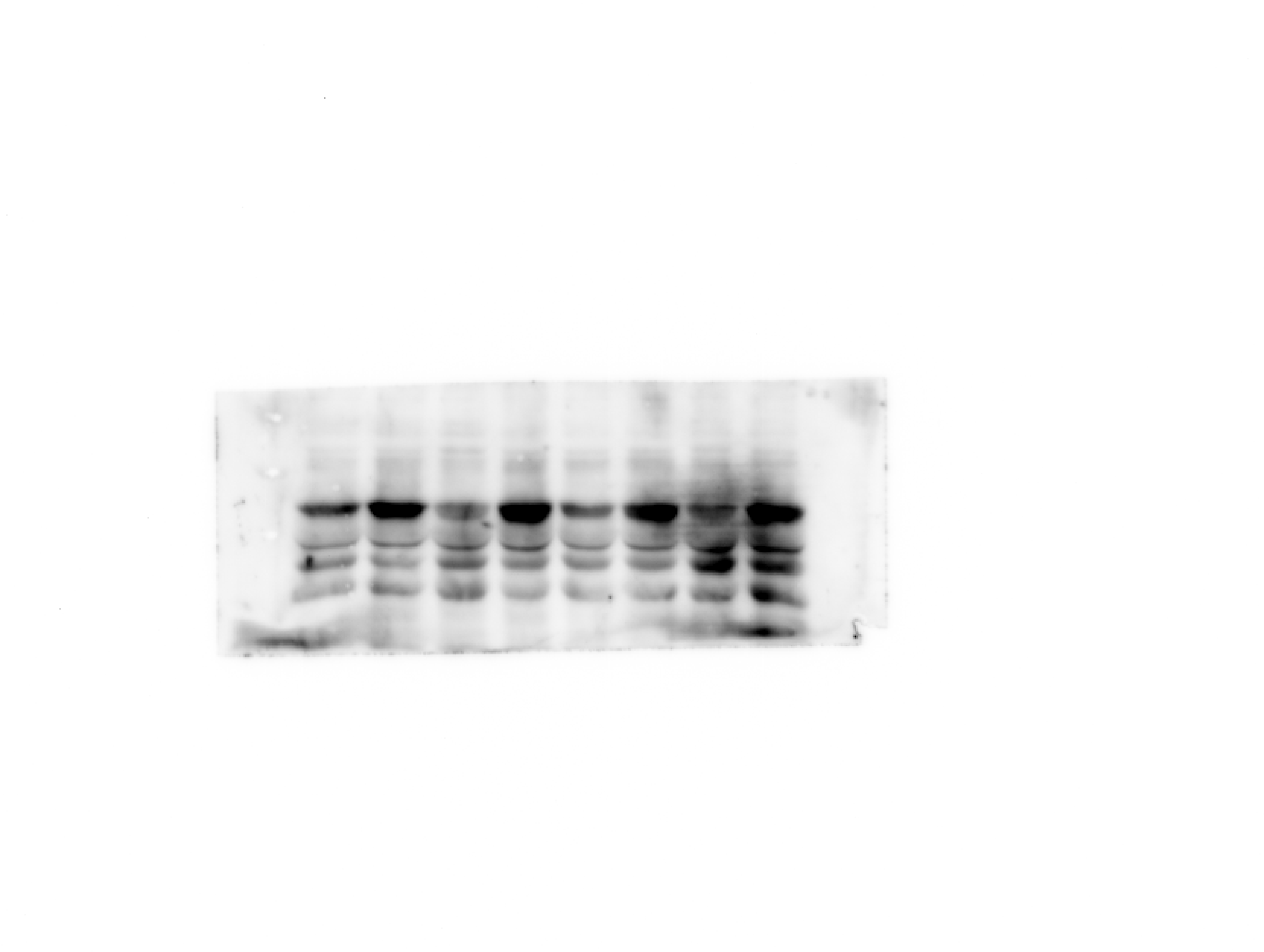

Supplement: Supplementary file 2 [file DataSheet1.ZIP › Westen blot/figure 2/3、GSK3β.tif]

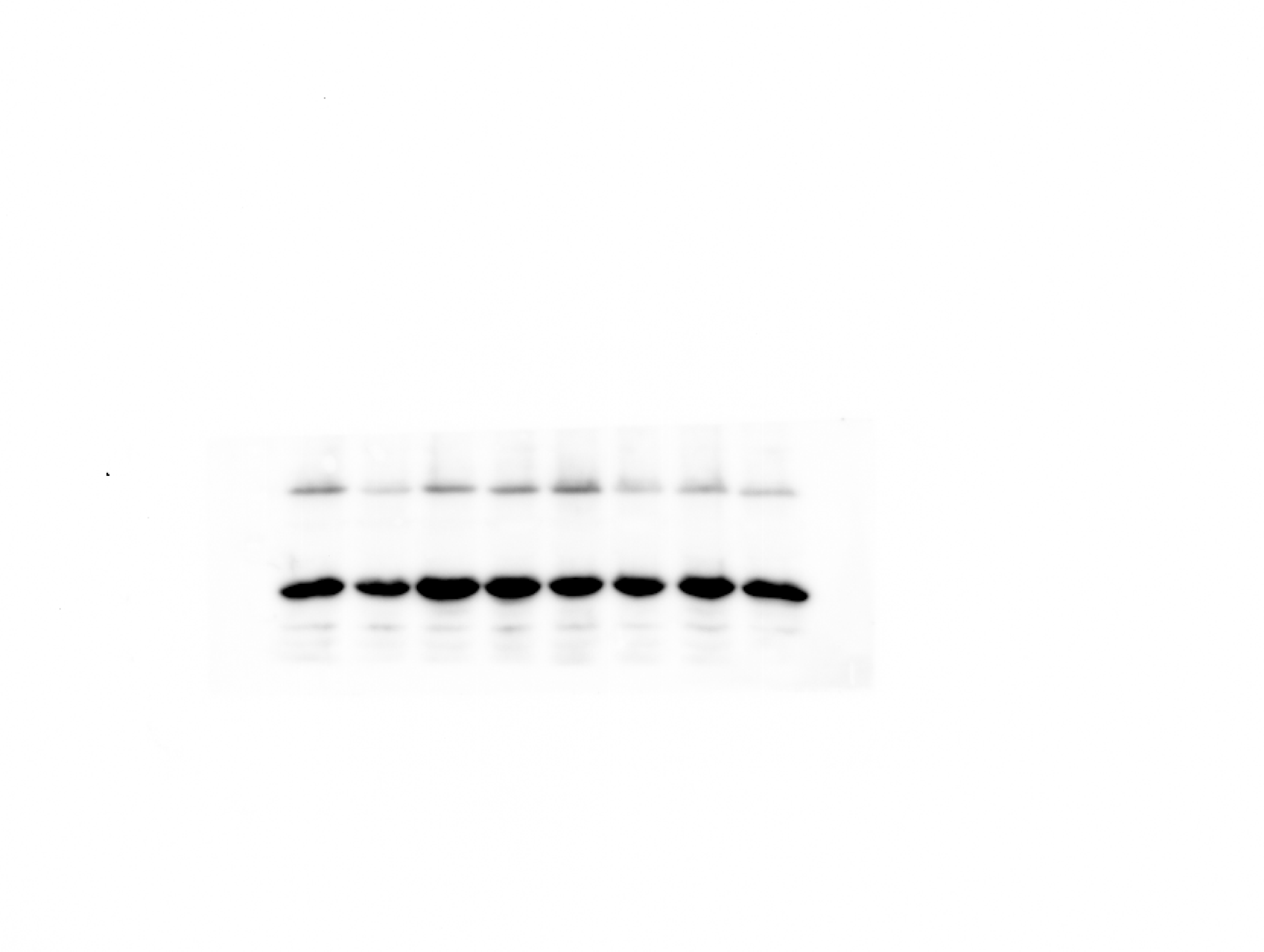

Supplement: Supplementary file 2 [file DataSheet1.ZIP › Westen blot/figure 2/4、GAPDH .tif]

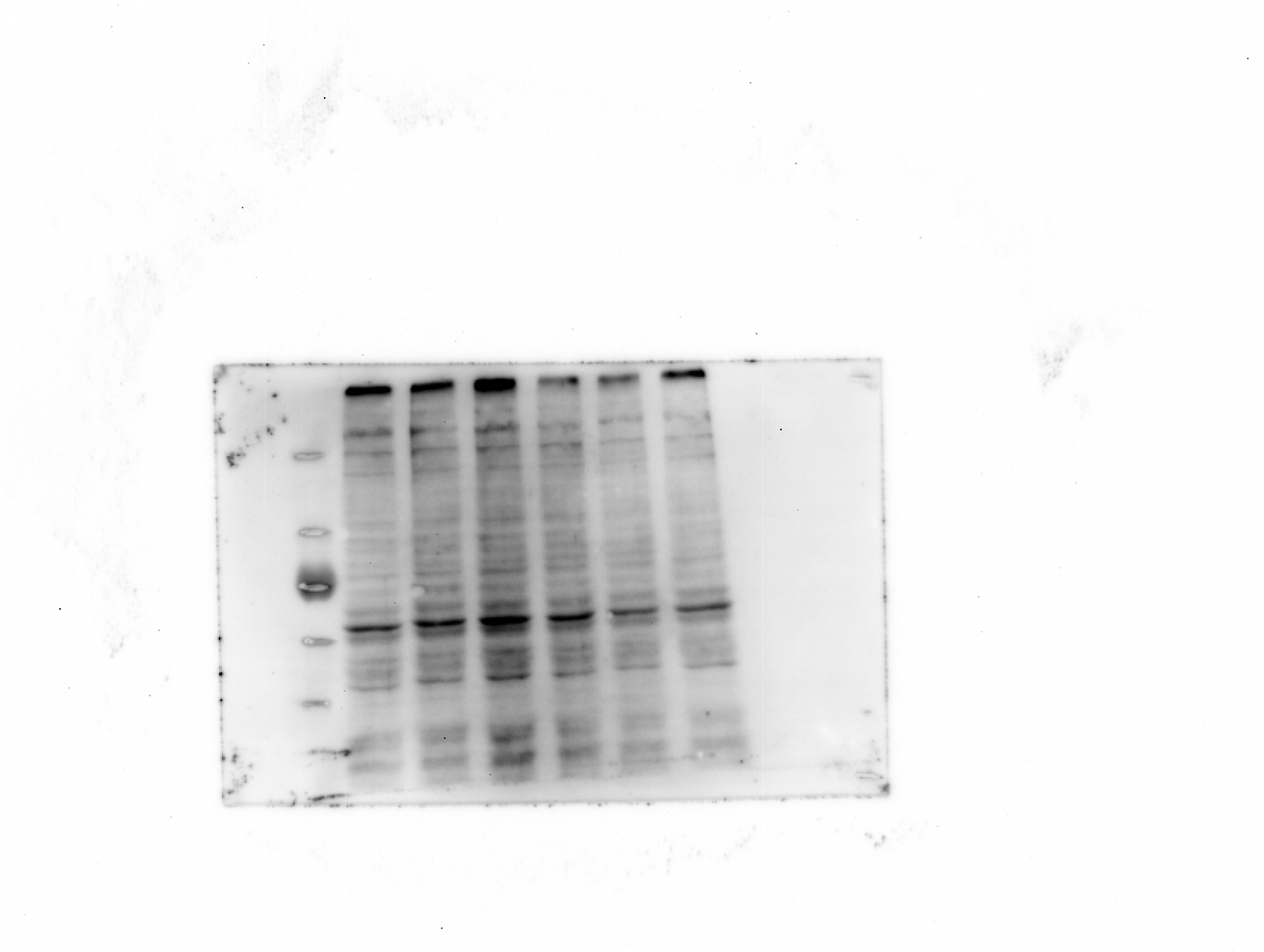

Supplement: Supplementary file 2 [file DataSheet1.ZIP › Westen blot/figure 3/C2C12/1、p-AKT.tif]

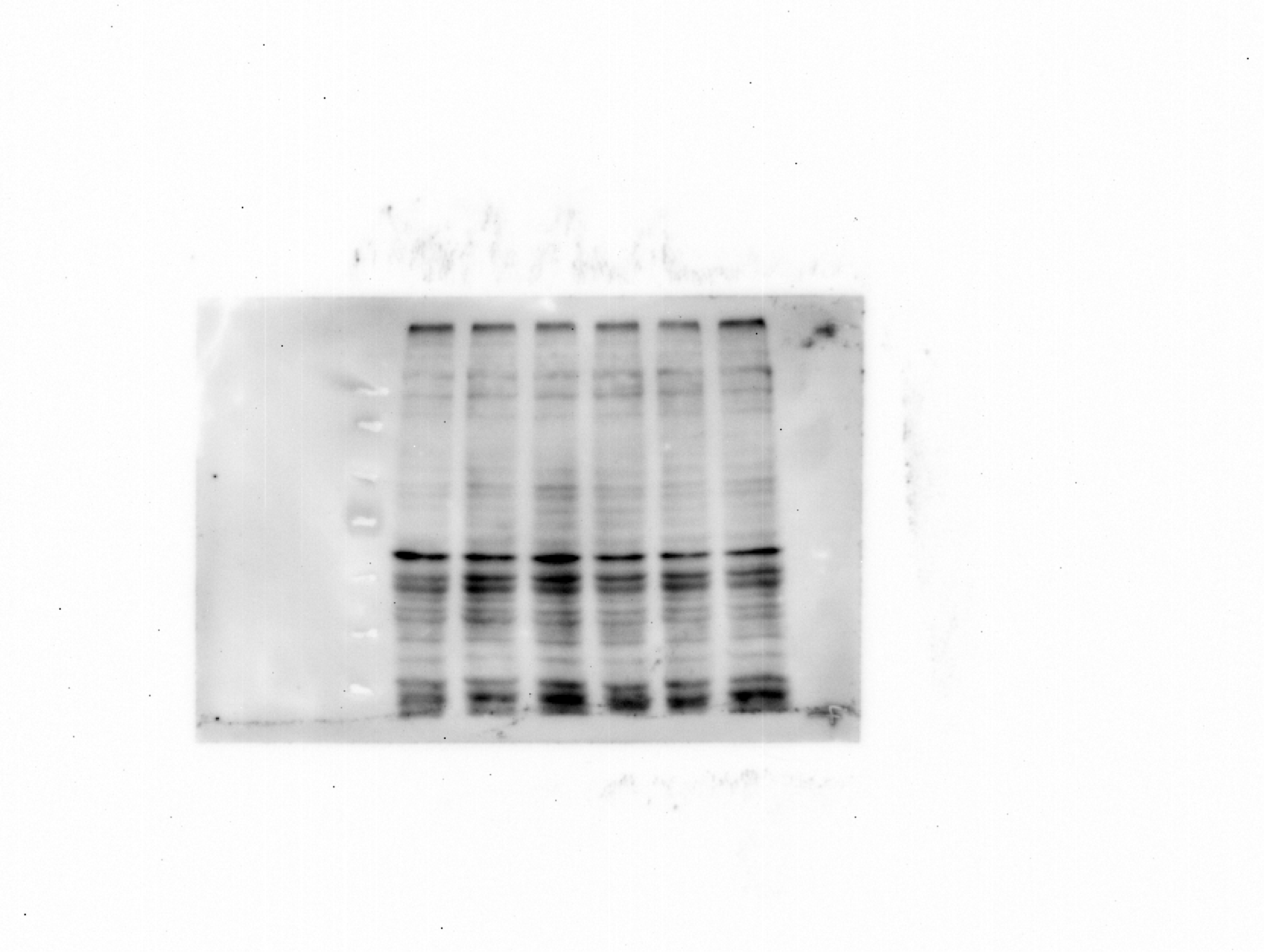

Supplement: Supplementary file 2 [file DataSheet1.ZIP › Westen blot/figure 3/C2C12/2、AKT.tif]

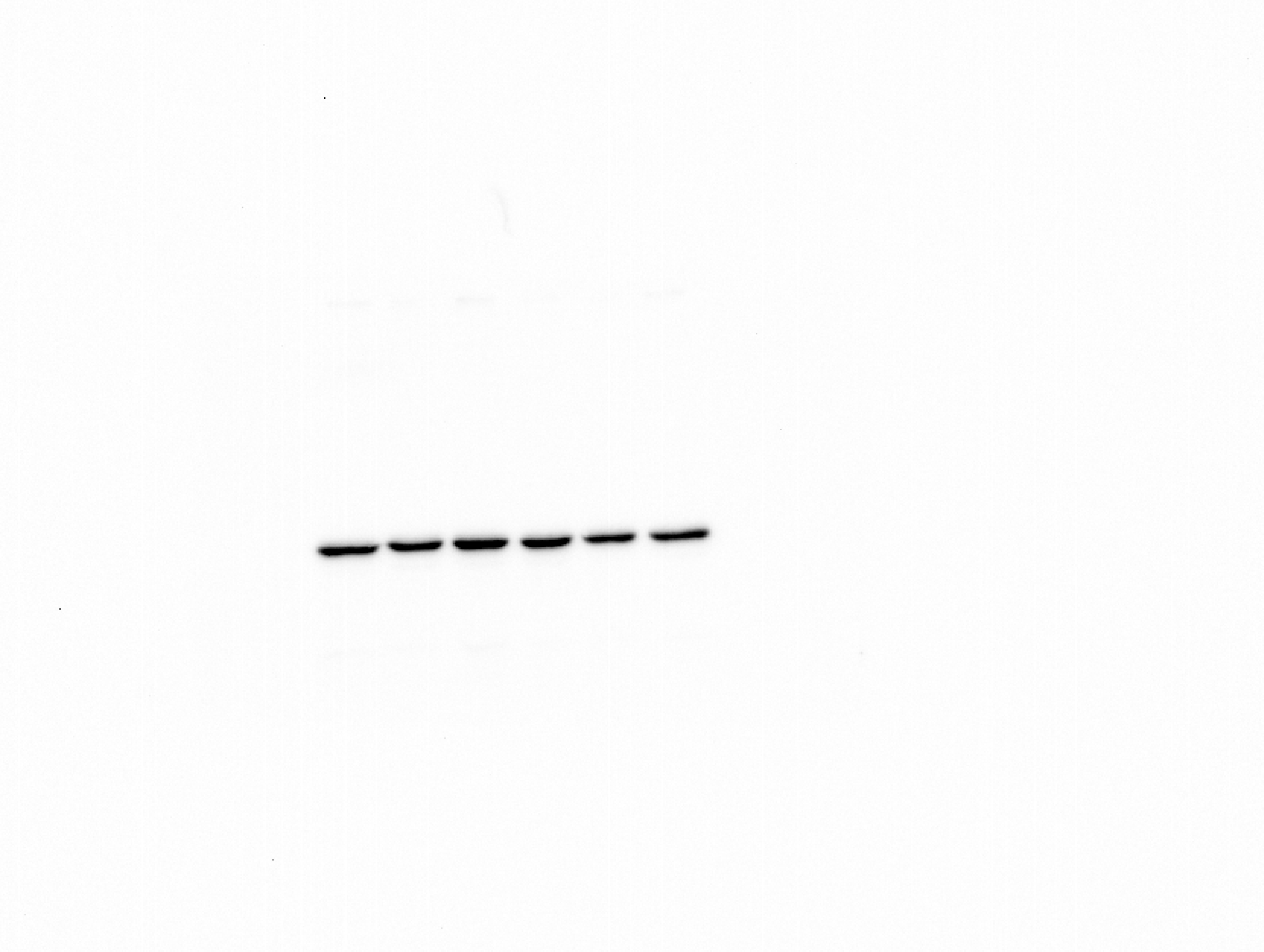

Supplement: Supplementary file 2 [file DataSheet1.ZIP › Westen blot/figure 3/C2C12/3、p-AKT AKT---β-tubulin.tif]

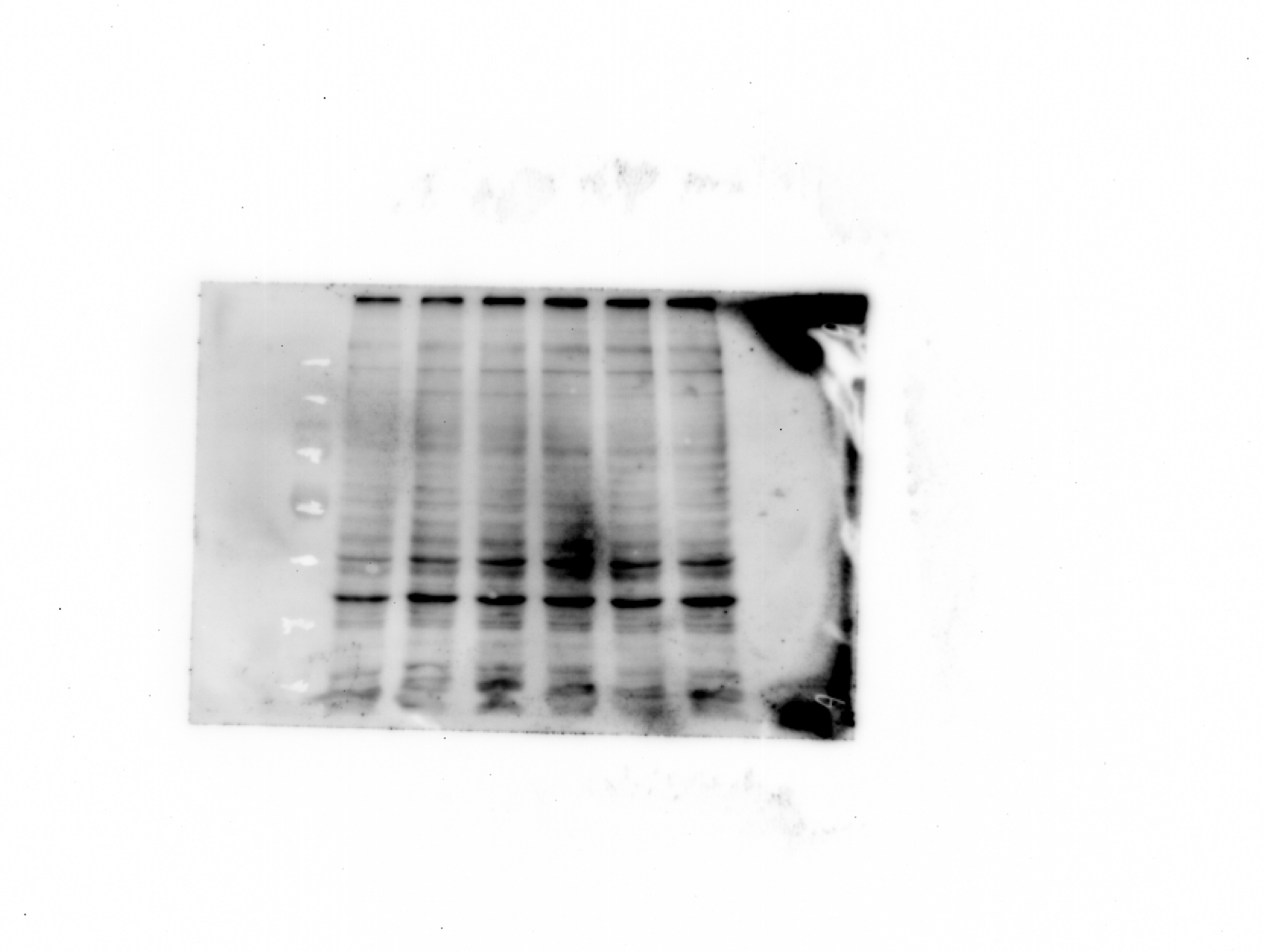

Supplement: Supplementary file 2 [file DataSheet1.ZIP › Westen blot/figure 3/C2C12/4、gsk3β.tif]

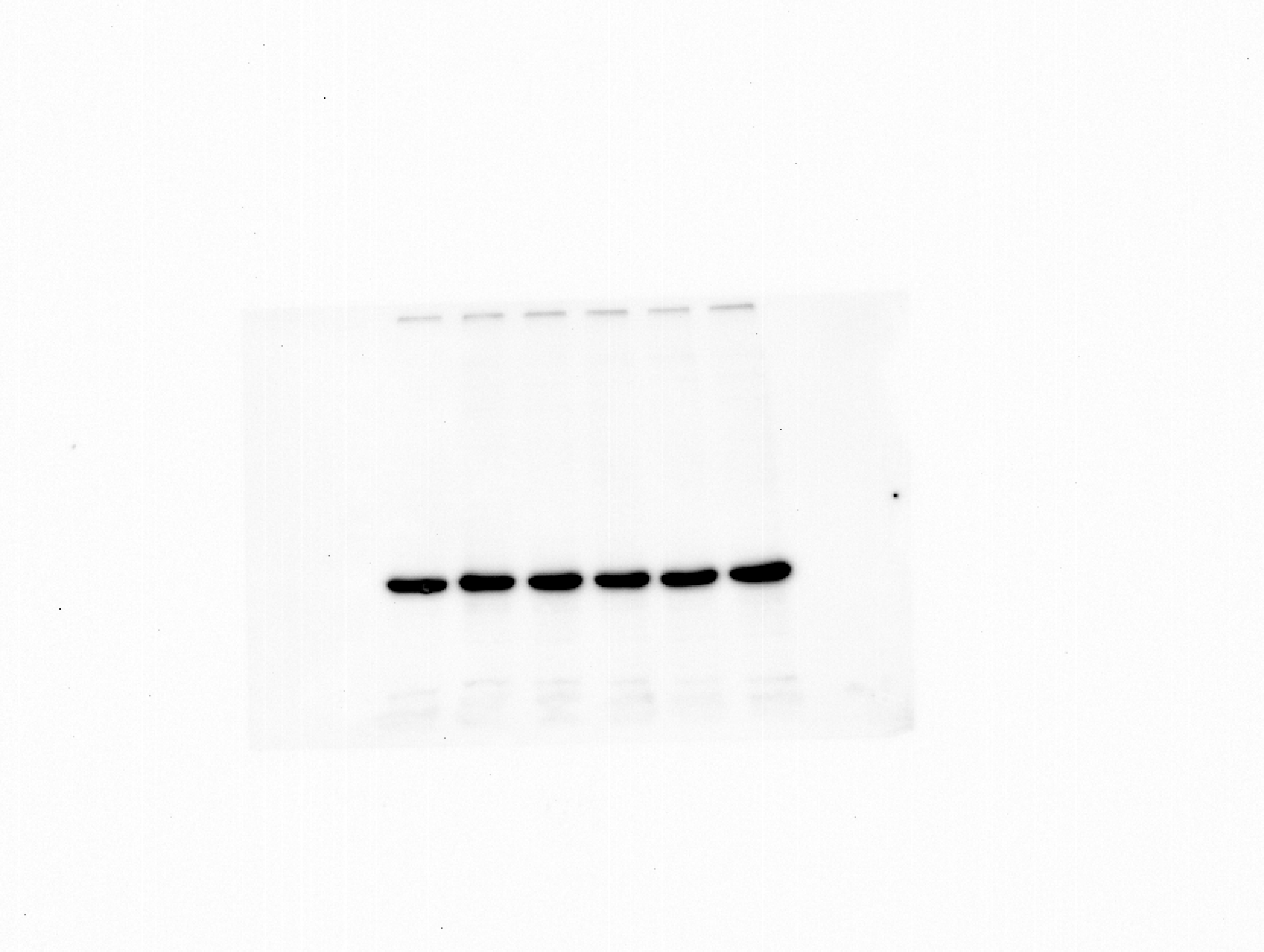

Supplement: Supplementary file 2 [file DataSheet1.ZIP › Westen blot/figure 3/C2C12/5、gsk3β--β-tubulin.tif]

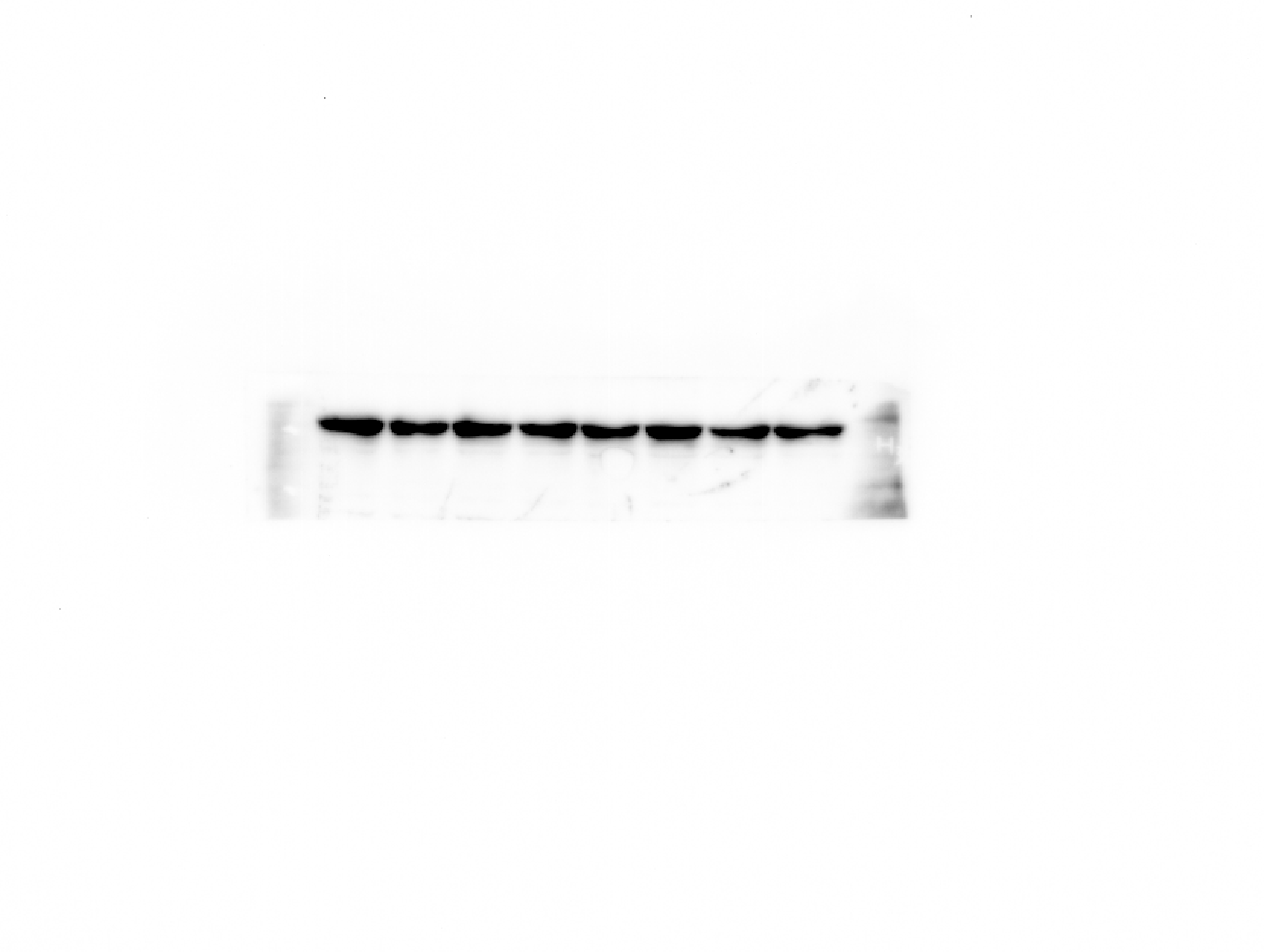

Supplement: Supplementary file 2 [file DataSheet1.ZIP › Westen blot/figure 3/HEPA/1、G6p.tif]

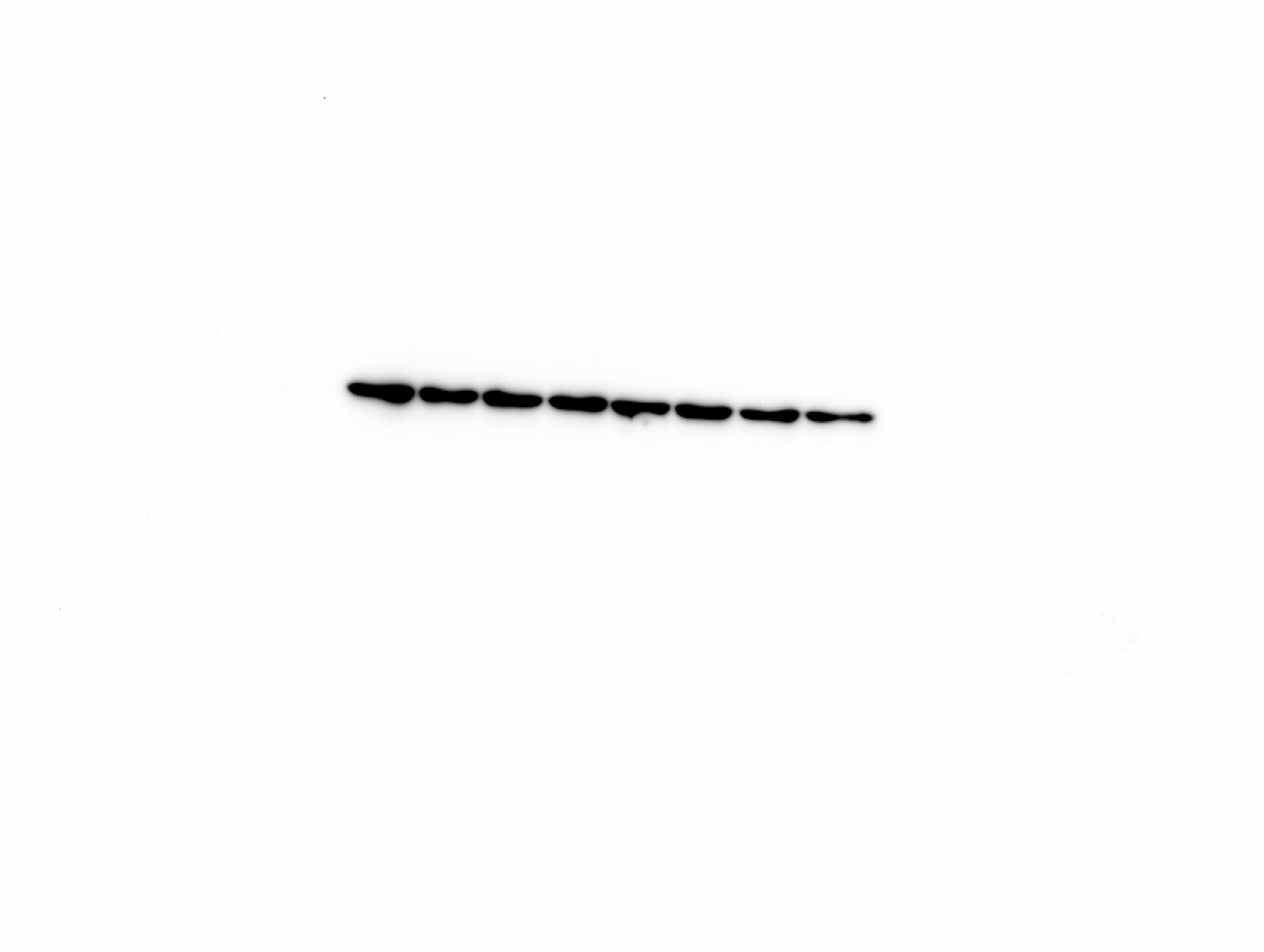

Supplement: Supplementary file 2 [file DataSheet1.ZIP › Westen blot/figure 3/HEPA/2、G6p-β-tubulin.tif]

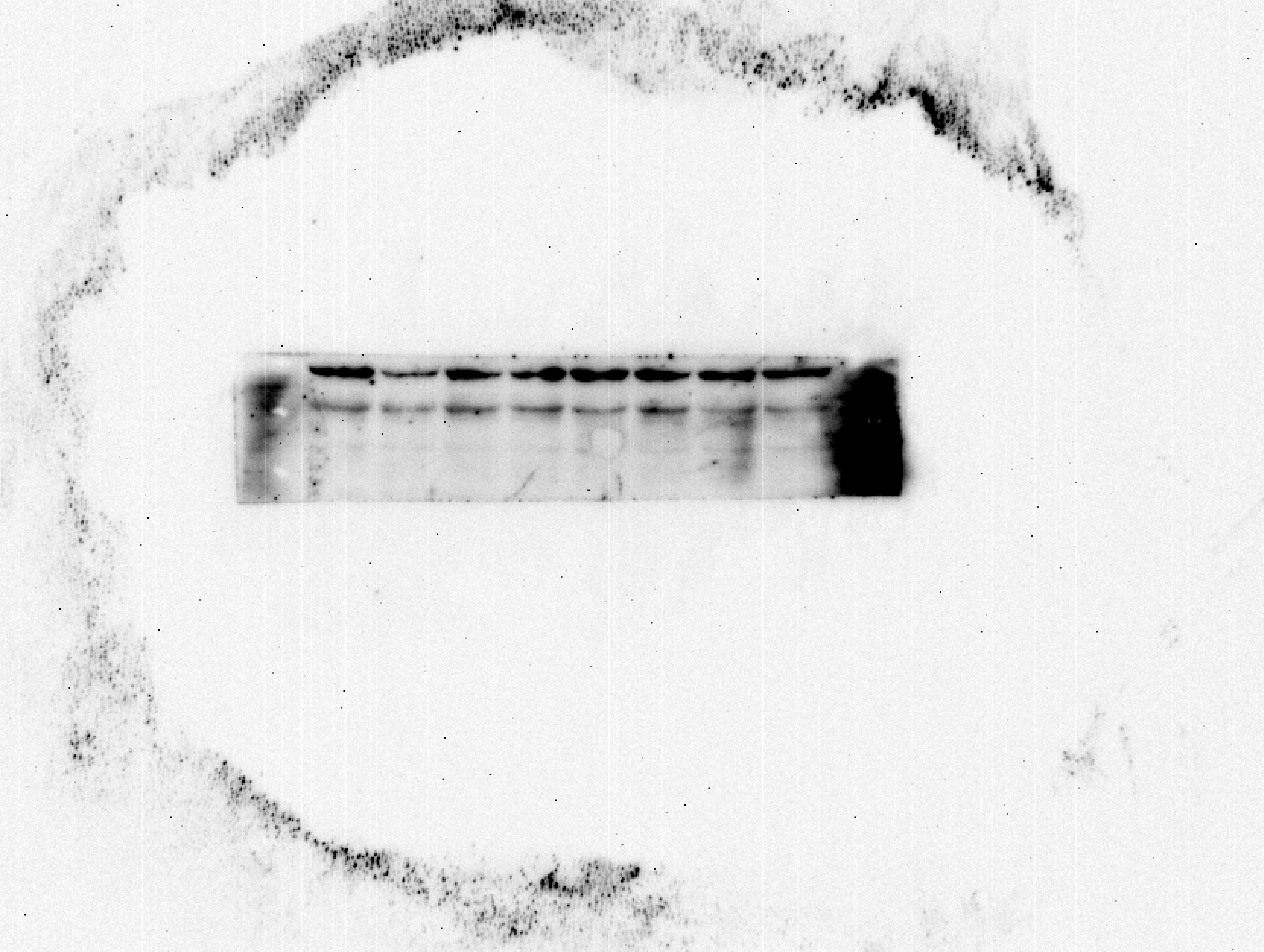

Supplement: Supplementary file 2 [file DataSheet1.ZIP › Westen blot/figure 3/HEPA/3、PEPCK.tif]

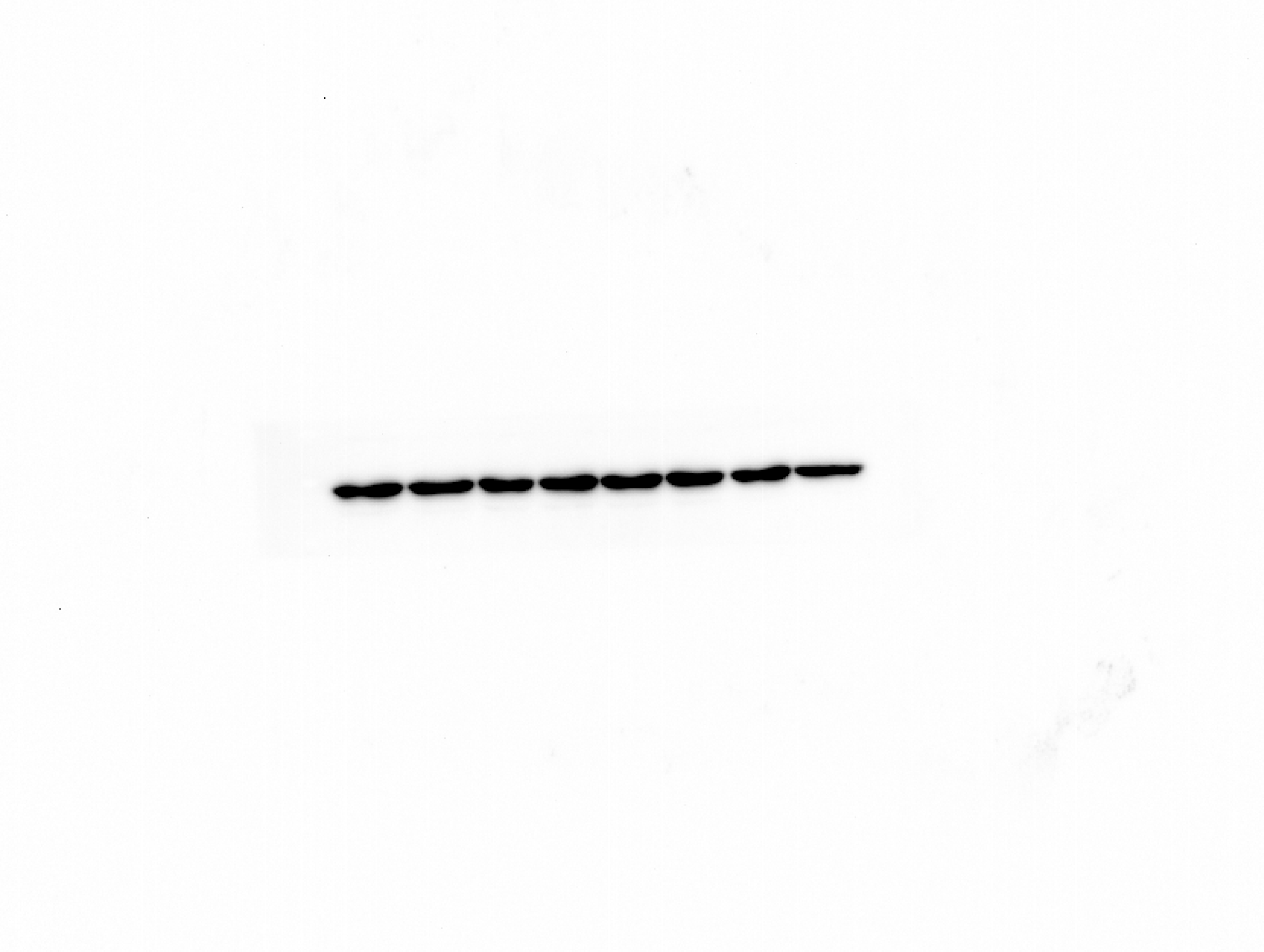

Supplement: Supplementary file 2 [file DataSheet1.ZIP › Westen blot/figure 3/HEPA/4、PEPCK-β-tubulin.tif]

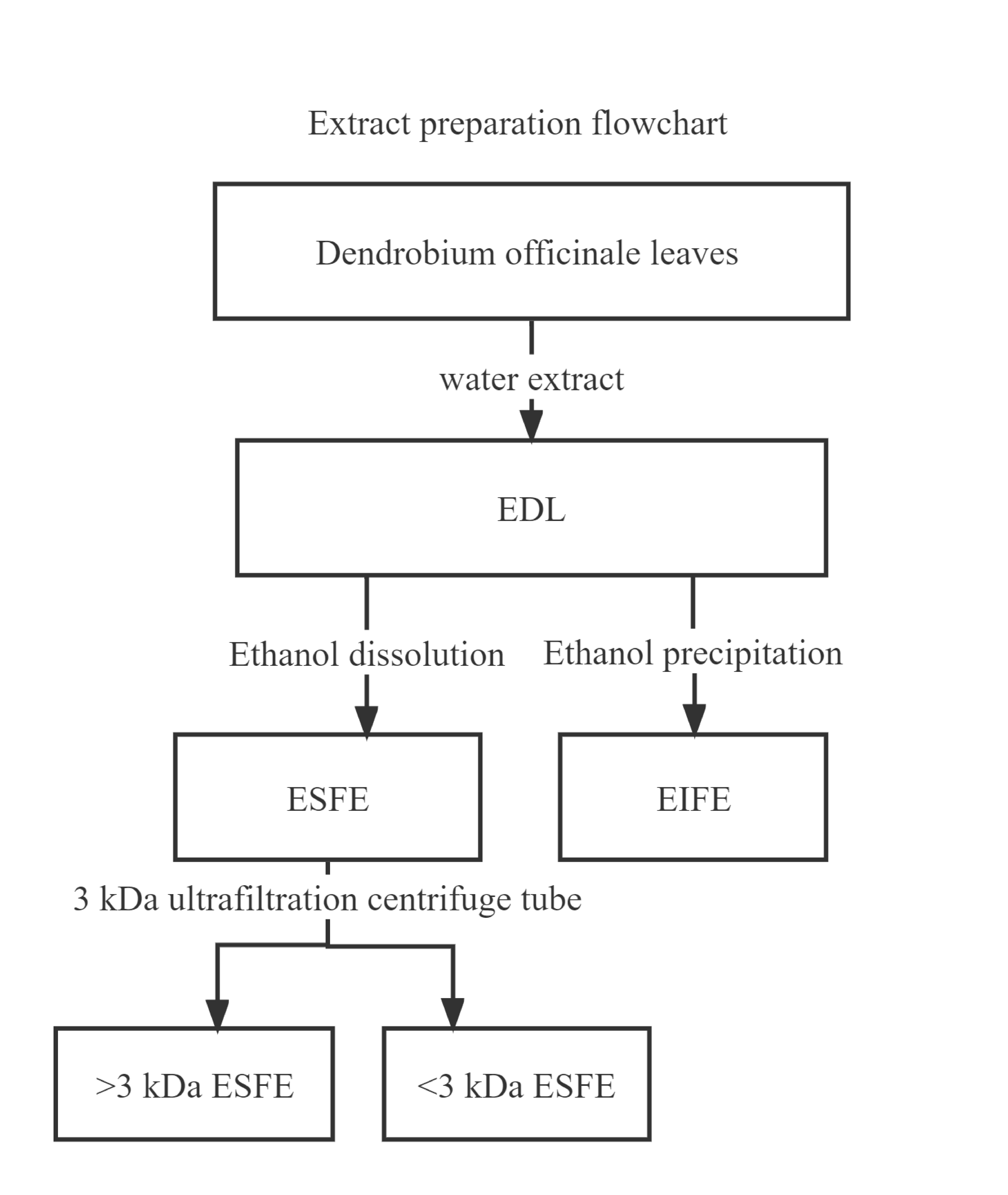

Supplement: Supplementary file 3 [file Image2.JPEG]
